# Improving accessibility and distinction between negative results in biomedical relation extraction

**DOI:** 10.5808/GI.2020.18.2.e20

**Published:** 2020-06-15

**Authors:** Diana Sousa, Andre Lamurias, Francisco M. Couto

**Affiliations:** LASIGE, Departamento de Informática, Faculdade de Ciências, Universidade de Lisboa, 1749-016 Lisboa, Portugal

**Keywords:** biomedical research, knowledge base, negative results, relation extraction

## Abstract

Accessible negative results are relevant for researchers and clinicians not only to limit their search space but also to prevent the costly re-exploration of research hypotheses. However, most biomedical relation extraction datasets do not seek to distinguish between a false and a negative relation among two biomedical entities. Furthermore, datasets created using distant supervision techniques also have some false negative relations that constitute undocumented/unknown relations (missing from a knowledge base). We propose to improve the distinction between these concepts, by revising a subset of the relations marked as false on the phenotype-gene relations corpus and give the first steps to automatically distinguish between the false (F), negative (N), and unknown (U) results. Our work resulted in a sample of 127 manually annotated FNU relations and a weighted-F1 of 0.5609 for their automatic distinction. This work was developed during the 6th Biomedical Linked Annotation Hackathon (BLAH6).

**Availability:** The code supporting our work and the sample of 127 manually annotated FNU relations of the PGR dataset is publicly available at https://github.com/lasigeBioTM/blah6.

## Introduction

Researchers and clinicians need to have access not only to known relations between biomedical entities but also to relations that were already disproven. Accessible negative results limit their search space and prevent the costly re-exploration of research hypotheses. However, most biomedical relation extraction datasets do not seek to distinguish between a false and a negative relation among two biomedical entities, and few knowledge bases hold negative examples. Some domain-specific exceptions are worth noticing, such as the Negatome database [1] for protein-protein interactions, and the phenotype-disease relations annotation file made available by the Human Phenotype Ontology (HPO) organization [2] that contains both positive and negative relations.

A false relation should express a context where the entities are not related. In contrast, a negative relation should express a context where there is an affirmation of no association between the two entities. Furthermore, datasets created using distant supervision techniques also have some false negative relations that constitute undocumented/unknown relations [3]. These relations are not marked true because they are not described in a knowledge base at the moment of the dataset creation, even though upon reading the context of these relations within their respective sentences one can support a true relation. Unknown relations are good examples of hypotheses to be further explored by researchers and clinicians and can be of use to effectively populate the biomedical relations knowledge bases.

We propose to improve the distinction between false, negative, and unknown (FNU) relations, by:

‒ Revising a subset of the relations marked as false on the phenotype-gene relations (PGR) corpus [4] to create a sample dataset of FNU relations (made available on PubAnnotation platform (http://pubannotation.org/collections/Annotation%20of%20Human%20Phenotype-Gene%20Relations%20-%20Identification%20of%20Negative,%20False,%20and%20Unknown%20Relations) [5])

‒ Implementing the first steps (using regular expressions and a neural network) to automatically distinguish between the FNU relations, using the previous sample FNU dataset as a test set.

## Methodology

**Fig. 1.**
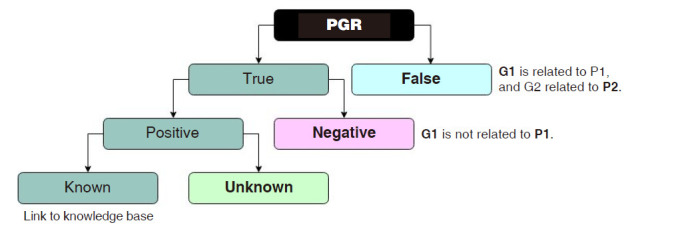
Illustration of the levels that correspond to the true phenotype-gene relations (PGR) relations (true, positive, and known), and false PGR relations (false, negative, and unknown). Also, some generic sentences that elucidate the distinction between false and negative relations, and the distinction between known and unknown relations, according to the authors.

The PGR corpus consists of 1,712 abstracts, 5,676 human phenotype annotations, 13,835 gene annotations, and 4,283 relations [4]. This automatically annotated corpus distinguishes between false and true relations but fails to identify different types of FNU relations. Fig. 1 illustrates the levels that we considered to represent true PGR relations (true, positive, and known), and false PGR relations (false, negative, and unknown).

Previously, our team had an expert curating a subset of the PGR corpus (around 30%). These annotations were initially divided into true, and false, for a different scope out of the reach of this work. Nonetheless, for this work, we used the 127 false annotations curated by our domain expert in that subset to make the distinction between false (F), negative (N), and unknown (U) relations. The distribution of each type of relation is displayed in Table 1.

**Fig. 2.**
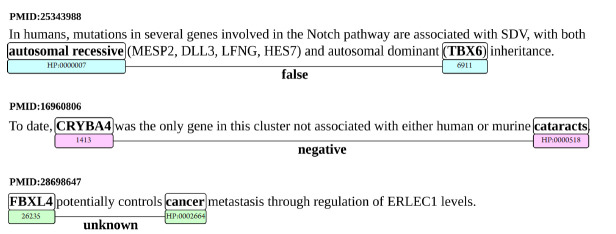
Example sentences for each type of false, negative, and unknown (FNU) relation: false (PMID:25343988), negative (PMID:16960806), and unknown (PMID:28698647). Also, the identified entities for each sentence, and their identifiers in the National Center for Biotechnology Information (NCBI) (for genes) and HPO (for human phenotypes).

Some concrete examples of what sentences constitute each type of relation are presented in Fig. 2.

The manual annotations allowed for the assessment of common patterns for the false and negative types of relations:

‒ False relations are often enumerations or an explanation of protocol that does not imply any type of relation.

‒ Negative relations are more regular, with words that imply the negation of association, such as *non, no, dissociation*, and *not*, frequently combined with *associated*, and *involved*.

Contrarily, unknown relations follow intractable patterns and are the most heterogeneous.

The first approach towards catching false and negative examples that follow the specified patterns was using regular expressions by:

‒ Analyzing the list of detected negative expressions and of detected false expressions and possible equivalences (for instance, for the negative expressions list, *not associated*).

‒ Introducing patterns that use those expressions, such as '('+gene_entity+'|'+phenotype_entity+')(.*?)'+negative_expression+'(.*?)('+gene_entity+'|'+phenotype_entity+')' that translates to *gene or phenotype followed by negative expression followed by gene or phenotype* (for negative examples).

‒ Evaluating using the manually curated dataset of 127 FNU relations (gold standard dataset) if those patterns are able to correctly classify the FNU relations.

Using regular expressions based on the annotation process can and probably will introduce a bias towards the relations that we annotated. Further applications of these regular expressions should be explored for the approach to be fully validated. Nevertheless, the creation of the regular expressions was done posteriorly to the annotation process, solely based on the patterns described above, with the goal of generalizing as much as possible to avoid overfitting.

As a second approach, we briefly tried to apply a neural network using the Keras library (without any tuning, due to time constraints). For this purpose, we divided the FNU dataset into a training set (70%, 89 FNU relations) and a test set (30%, 38 FNU relations).

## Results and Discussion

The application of a small subset of regular expressions to catch false and negative examples that follow the previously mentioned patterns had some promising results. We opted for the unknown relation as our default label since this type of sentences are more heterogeneous with irregular patterns that are difficult to capture by the use of regular expressions. Testing against the gold standard dataset shows a weighted-F1 of 0.5609. Other relevant metrics are displayed in Table 2.

The use of the neural network produced poor results (0.2308 accuracy) mainly due to the lack of tuning and the small size of our FNU dataset.

These preliminary results show that it is possible to capture common patterns of false and negative relations with high precision, but also shows the need for more work and possible exploration of machine learning techniques in order to capture more instances of those types of relations. More manual work, building regular expressions, should boost these preliminary results. Using syntax and dependency parsing to capture complex enumerations can also boost performance (e.g., enumerations where a group of genes is associated with a phenotype A and another group of genes is related to phenotype B).

## Conclusions and Future Work

This work demonstrated that regular expressions are a feasible way of capturing differences between FNU relations, at least at a preliminary stage. The false and negative types of relations follow distinctive patterns that should be further explored to boost the weighted-F1 of 0.5609. Preliminary work with neural networks showed poor results (due to time constraints), but tuning the training and a larger dataset should boost these early results.

Future work could be revising all the false relations within the PGR corpus, and also of other datasets. Negative relations in manually annotated datasets should be easier to detect since the unknown relations would not be present. All of this will allow us to further explore machine learning approaches to tackle this problem more effectively.

## Figures and Tables

**Table 1.** Distribution of each type of FNU relation: false, negative, unknown, and the total number of relations

	False	Negative	Unknown	Total
No.	73	11	43	127

**Table 2.** The evaluation metrics (precision, recall, and f-measure) for the false, negative, and unknown relations, and the weighted-F1 for all classes

Type of relation	Precision	Recall	F-measure	Weighted-F1
False	0.8438	0.3699	0.5143	0.5609
Negative	0.8333	0.4545	0.5882	
Unknown	0.427	0.8837	0.5758	
